# Application of deep learning model based on unenhanced chest CT for opportunistic screening of osteoporosis: a multicenter retrospective cohort study

**DOI:** 10.1186/s13244-024-01817-2

**Published:** 2025-01-10

**Authors:** Chengbin Huang, Dengying Wu, Bingzhang Wang, Chenxuan Hong, Jiasen Hu, Zijian Yan, Jianpeng Chen, Yaping Jin, Yingze Zhang

**Affiliations:** 1 Department of Orthopaedics, The Second Affiliated Hospital and Yuying Children’s Hospital of Wenzhou Medical University, Wenzhou, Zhejiang Province China; 2 Department of Orthopaedics, Wenzhou Hospital of Integrated Traditional Chinese and Western Medicine, Wenzhou, Zhejiang Province China; 3 Department of Orthopaedics, People’s Hospital of Cangnan, Wenzhou, Zhejiang Province China; 4 Department of Orthopaedics, Yueqing People’s Hospital, Yueqing, Zhejiang Province China; 5 School of Medicine, Nankai University, Tianjin, China; 6 Department of Orthopaedics, The Third Hospital of Hebei Medical University, Shijiazhuang, Hebei Province China

**Keywords:** Osteoporosis, Chest CT, Deep learning, Convolutional neural network, Multicenter cohort study

## Abstract

**Introduction:**

A large number of middle-aged and elderly patients have an insufficient understanding of osteoporosis and its harm. This study aimed to establish and validate a convolutional neural network (CNN) model based on unenhanced chest computed tomography (CT) images of the vertebral body and skeletal muscle for opportunistic screening in patients with osteoporosis.

**Materials and methods:**

Our team retrospectively collected clinical information from participants who underwent unenhanced chest CT and dual-energy X-ray absorptiometry (DXA) examinations between January 1, 2022, and December 31, 2022, at four hospitals. These participants were divided into a training set (*n* = 581), an external test set 1 (*n* = 229), an external test set 2 (*n* = 198) and an external test set 3 (*n* = 118). Five CNN models were constructed based on chest CT images to screen patients with osteoporosis and compared with the SMI model to predict the performance of osteoporosis patients.

**Results:**

All CNN models have good performance in predicting osteoporosis patients. The average F1 score of Densenet121 in the three external test sets was 0.865. The area under the curve (AUC) of Desenet121 in external test set 1, external test set 2, and external test set 3 were 0.827, 0.859, and 0.865, respectively. Furthermore, the Densenet121 model demonstrated a notably superior performance compared to the SMI model in predicting osteoporosis patients.

**Conclusions:**

The CNN model based on unenhanced chest CT vertebral and skeletal muscle images can opportunistically screen patients with osteoporosis. Clinicians can use the CNN model to intervene in patients with osteoporosis and promptly avoid fragility fractures.

**Critical relevance statement:**

The CNN model based on unenhanced chest CT vertebral and skeletal muscle images can opportunistically screen patients with osteoporosis. Clinicians can use the CNN model to intervene in patients with osteoporosis and promptly avoid fragility fractures.

**Key Points:**

- The application of unenhanced chest CT is increasing.
- Most people do not consciously use DXA to screen themselves for osteoporosis.
- A deep learning model was constructed based on CT images from four institutions.

**Graphical Abstract:**

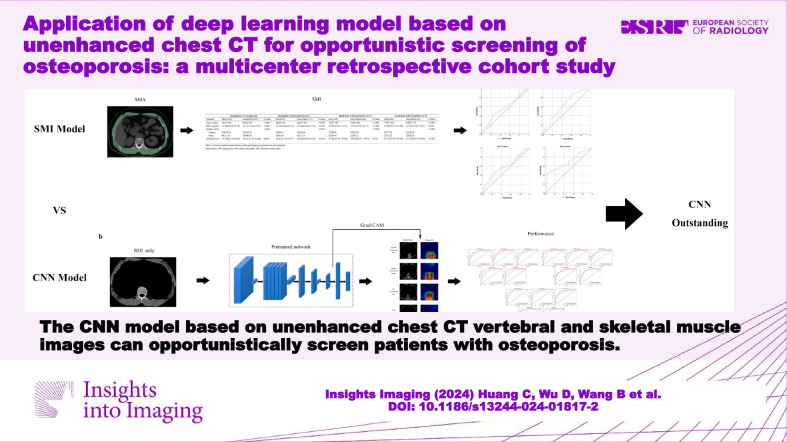

## Introduction

Osteoporosis (OP) is a common bone disease resulting in reduced bone density and an increased fracture risk. Osteoporosis can occur at any age but is more common in postmenopausal women and older men [1, 2]. As the global population continues to age, the prevalence of osteoporosis is escalating rapidly, emerging as a prominent public health concern. Among the Chinese population over 50, 29.0% of women and 13.5% of men have osteoporosis, accounting for 49 million and 22.8 million respectively [3]. Fractures resulting from osteoporosis stand as a prominent cause of disability and mortality among elderly individuals [4]. Within one year of hip fracture, 33% of patients may die from complications [5]. Therefore, the early diagnosis and treatment of osteoporosis is essential. Bone mineral density (BMD) measured by dual-energy X-ray absorptiometry (DXA) is the gold standard for the diagnosis of osteoporosis [4]. However, the public’s awareness of osteoporosis and its dangers needs to be improved, and they do not consciously seek a doctor’s diagnosis of osteoporosis.

Sarcopenia is a condition characterized by the decline in muscle mass and strength commonly associated with the aging process [6]. Sarcopenia and osteoporosis often co-exist, and sarcopenia can increase the risk of osteoporosis [7]. Studies by Lima et al [8] show that the incidence of osteoporosis in non-sarcopenia patients is much lower than in patients with severe sarcopenia. In addition, computed tomography (CT)-based skeletal muscle index (SMI) can diagnose sarcopenia and predict osteoporosis patients to a certain extent [9, 10].

As computer computing power advances swiftly, artificial intelligence technology is progressively finding broader applications in the field of medicine [11]. In image recognition, artificial intelligence is mainly divided into two fields: radiomics and deep learning. Research indicates that radiomic models based on CT vertebral body or muscle images can predict osteoporosis patients well [12, 13]. However, radiomics has shortcomings, such as complex preprocessing, and limited feature extraction Deep learning, especially convolutional neural network (CNN) models, can learn automatically from large amounts of training data, and their diagnostic accuracy can match or even exceed that of human doctors. For example, applying the CNN model based on spinal X-rays can be a good predictor of osteoporosis patients [14].

CT examinations have exponentially increased in recent years, especially unenhanced chest CT. Middle-aged and older people get chest CT for various reasons, such as disease screening, monitoring, or preoperative preparation. Therefore, this study intends to make secondary use of unenhanced chest CT images and construct a CNN model based on muscle and vertebral body images from multiple institutions to screen patients with osteoporosis.

## Materials and methods

### Study design

This study retrospectively collected clinical data of patients from the Second Affiliated Hospital of Wenzhou Medical University (Institution 1, Training set), Yueqing People’s Hospital (Institution 2, External test set 1), Wenzhou Hospital of Integrated Traditional Chinese and Western Medicine (Institution 3, External test set 2) and People’s Hospital of Cangnan (Institution 4, External test set 3) from January 1, 2022 to December 31, 2022, respectively. Moreover, all institutions are tertiary hospitals. The institutional review boards at all participating institutions granted approval for this retrospective multicohort study (approval number: 2023-K-179-01) and waived the requirement for written informed consent.

**Fig. 1:**
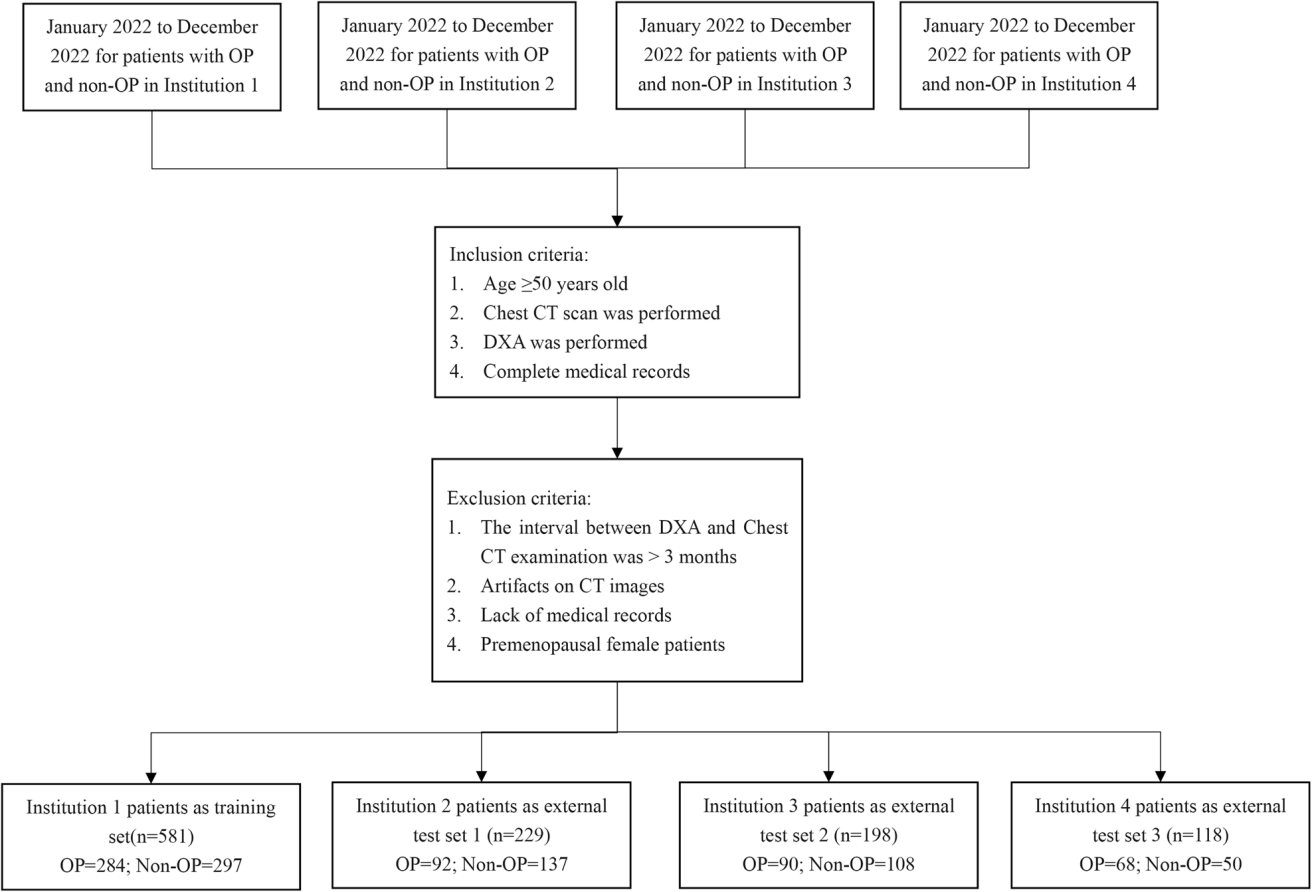
The recruitment process of participants in this study. CT, computed tomography; DXA, dual-energy X-ray absorptiometry; OP, osteoporosis

A total of 1126 participants from four institutions were included in the study. The inclusion criteria for this study were (1) age ≥ 50 years, (2) Unenhanced chest CT scan was performed, (3) DXA was performed, and (4) Complete medical records. The exclusion criteria for this study were (1) The interval between DXA and chest CT examination was > 3 months, (2) Artifacts on CT images, (3) Lack of medical records, and (4) Premenopausal female participants. Institution 1 registered 581 participants, comprising 284 with osteoporosis and 297 without osteoporosis. Institution 2 had 229 participants, with 92 having osteoporosis and 137 without osteoporosis. Institution 3 enrolled 198 participants, with 90 having osteoporosis and 108 without osteoporosis. Institution 4 included 118 participants, with 68 having osteoporosis and 50 without osteoporosis. The participants recruitment process for this study is shown in Fig. 1.

DXA is considered the gold standard for diagnosing osteoporosis through the measurement of BMD. For postmenopausal women and men (over 50 years old), referring to the diagnostic criteria is recommended. Patients with a t score of −2.5 or less on any lumbar spine (lumbar 1–4), femoral neck, or distal radius thirds measured by DXA were diagnosed as osteoporosis patients. Patients with a t score greater than –2.5 were diagnosed as non-osteoporosis patients.

Unenhanced chest CT images of 5 mm thickness of Institution 1, Institution 2, Institution 3, and Institution 4 were obtained from picture archiving and communication systems (PACS), respectively. In addition, all CT images were resampled to a voxel spacing of 1 × 1 × 1 mm^3^ in order to normalize the images from different institutions. At the same time, adjust the window level of all images to 250 and the window width to 800.

### Ethics approval and consent to participate

The Ethics Committee of the Second Affiliated Hospital of Wenzhou Medical University, the Ethics Committee of Yueqing People’s Hospital, Wenzhou Hospital of Integrated Traditional Chinese and Western Medicine, and the People’s Hospital of Cangnan approved this study (approval number: 2023-K-179-01; YQYY202400061; 2024-L047; 2023120). Informed consent was waived because the study was a retrospective cohort study.

### Region of interesting

According to previous studies, the threshold of skeletal muscle is −29HU to 150HU [9]. A junior clinician (5 + years of clinical experience) used 3D Slicer (version 5.2.2) software to map the thoracic vertebrae and its surrounding muscles, the region of interest (ROI), at the mid-level of the 12th thoracic vertebrae (T12). In addition, the ROI outlined by the junior clinician was rechecked by a senior clinician (10 + years of clinical work experience). All clinicians are blind to patient information when mapping and checking ROI.

### SMI model construction

**Fig. 2:**
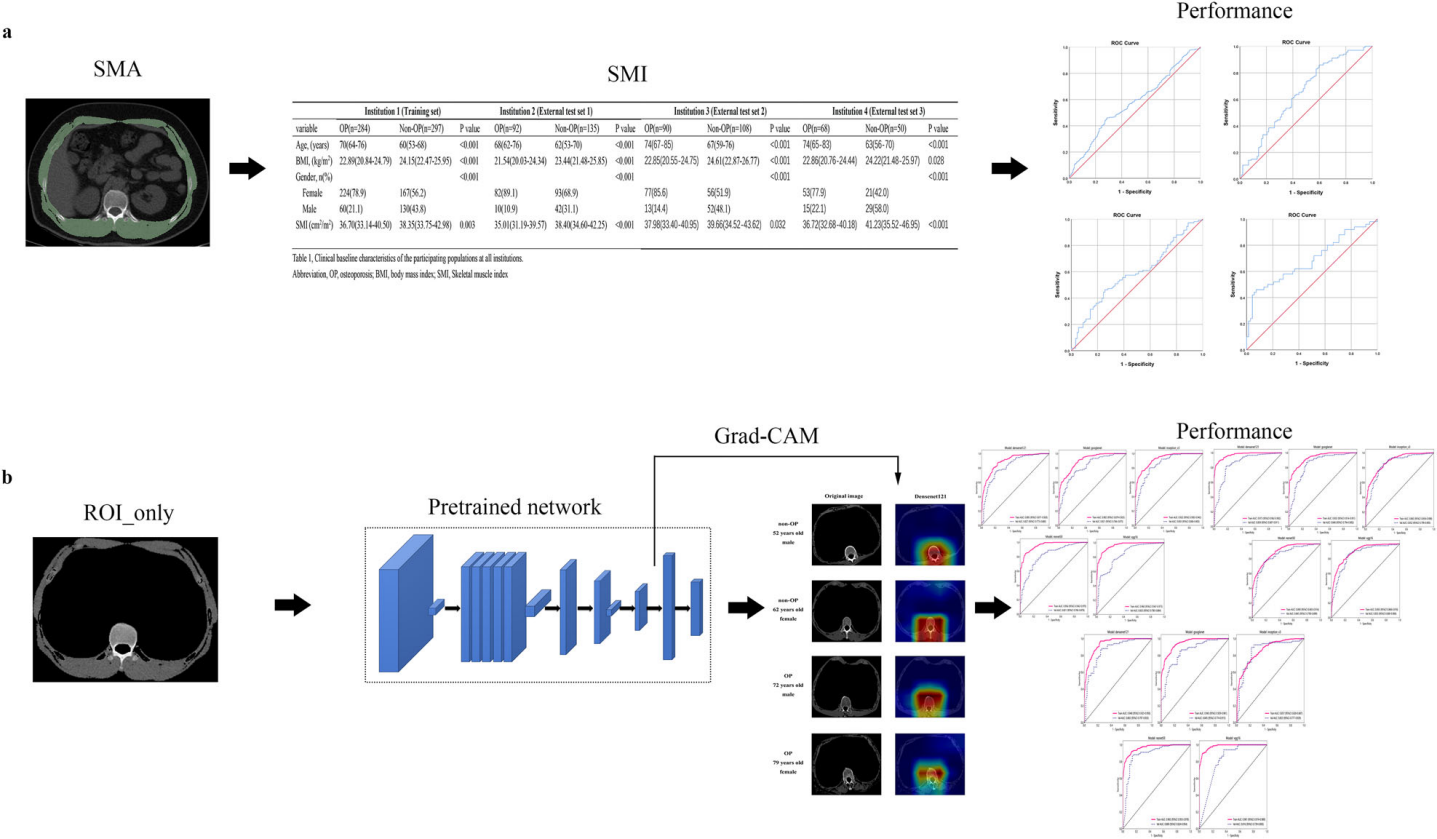
The research process of this study. **a** The process of building the SMI model. **b** The process of building CNN models. SMA, skeletal muscle area; SMI, skeletal muscle index; ROI, region of interesting; Grad-CAM, gradient-weighted class activation mapping; CNN, convolutional neural network

This study used ImageJ (NIH ImageJ version 1.54f) to calculate the area of the bilateral psoas muscle in ROI. The obtained skeletal muscle area (cm^2^) is divided by the square of the patient’s height (m^2^) to calculate SMI (cm^2^/m^2^). With the presence or absence of osteoporosis as a state variable, the receiver operator characteristic (ROC) curve was used to analyze the efficacy of SMI in predicting osteoporosis. Figure 2a summarizes this part of the process.

### CNN model construction

In the T12 vertebral plane CT images, only the ROI was isolated by cropping, with non-ROI areas such as the spinal cord and lungs being excluded. These ROI images were subsequently utilized for training and validating the CNN model. The data set of Institution 1 is the training set, and the data set of Institution 2, Institution 3, and Institution 4 is the external test set 1, external test set 2, and external test set 3, respectively. In this study, five CNN models (Densent121, Inception_v3, Googlenet, Resnet50, and VGG16) are constructed. Moreover, all models were pre-trained in the ImageNet dataset. The training process encompasses both forward computation and backpropagation. Before training, the input ROI was resized to 299 × 299 pixels for Inception_v3 and 224 × 224 pixels for the other CNN models, respectively. A stochastic gradient descent (SGD) optimizer was utilized to update the model parameters, with an initial learning rate of 0.01, which was adaptively adjusted using the cosine annealing algorithm. The training process extended over 200 epochs, with a fixed batch size of 32.

In addition, we use the gradient-weighted class activation mapping (Grad-CAM) [15] technique to visualize the last convolutional layer in the CNN model. Grad-CAM enables us to better understand the critical content of deep learning models to make decision recognition image information. Figure 2b summarizes this part of the process.

### Statistical analysis

The distribution of clinical baseline characteristic data was evaluated using the Shapiro-Wilk test. Patient characteristics were expressed as mean ± standard deviation for continuous variables and percentages for categorical variables. Continuous variables with a normal distribution underwent analysis using the Student’s *t*-test, whereas categorical variables were presented as percentages and analyzed using the Pearson Chi-square test. Performance evaluation of various models involved metrics such as sensitivity, specificity, accuracy, F1-score, and the area under the ROC curve. Model comparisons were conducted using the DeLong test on the ROC curves [16]. All data were processed using SPSS (version 26.0; SPSS et al, USA) and Python (version 3.9.7).

## Results

### Clinical baseline characteristics and SMI model performance

The study included 1126 participants: 581 in Institution 1, 229 in Institution 2, 198 in Institution 3, and 118 in Institution 4. Institution 1 included 284 patients with osteoporosis and 297 patients without osteoporosis; Institution 2 included 92 patients with osteoporosis and 137 patients without osteoporosis; Institution 3 included 90 patients with osteoporosis and 108 patients without osteoporosis; Institution 4 included 68 patients with osteoporosis and 50 patients without osteoporosis (Fig. 1). There were significant differences (*p*-values < 0.05) in age, gender, body mass index (BMI), and SMI between the osteoporosis and non-osteoporosis patient groups at all Institutions. The BMI and SMI of non-osteoporosis patients were higher than those of osteoporosis patients, and the proportion of female patients and age was lower than that of osteoporosis patients (details are shown in Table 1).

**Table 1:** Clinical baseline characteristics of the participating populations at all institutions

Institution 1 (training set)				Institution 2 (external test set 1)				Institution 3 (external test set 2)			Institution 4 (external test set 3)	
Variable	OP (*n* = 284)	Non-OP(*n* = 297)	*p*-value	OP (*n* = 92)	Non-OP (*n* = 135)	*p-*value	OP (*n* = 90)	Non-OP (*n* = 108)	*p-*value	OP (*n* = 68)	Non-OP (*n* = 50)	*p-*value
Age, (years)	70 (64–76)	60 (53–68)	< 0.001	68 (62–76)	62 (53–70)	< 0.001	74 (67–85)	67 (59–76)	< 0.001	74 (65–83)	63 (56–70)	< 0.001
BMI, (kg/m^2^)	22.89 (20.84–24.79)	24.15 (22.47–25.95)	< 0.001	21.54 (20.03–24.34)	23.44 (21.48–25.85)	< 0.001	22.85 (20.55–24.75)	24.61 (22.87–26.77)	< 0.001	22.86 (20.76–24.44)	24.22 (21.48–25.97)	0.028
Gender, n (%)			< 0.001			< 0.001			< 0.001			< 0.001
Female	224 (78.9)	167 (56.2)		82 (89.1)	93 (68.9)		77 (85.6)	56 (51.9)		53 (77.9)	21 (42.0)	
Male	60 (21.1)	130 (43.8)		10 (10.9)	42 (31.1)		13 (14.4)	52 (48.1)		15 (22.1)	29 (58.0)	
SMI (cm^2^/m^2^)	36.70 (33.14–40.50)	38.35 (33.75–42.98)	0.003	35.01 (31.19–39.57)	38.40 (34.60–42.25)	< 0.001	37.98 (33.40–40.95)	39.66 (34.52–43.62)	0.032	36.72 (32.68–40.18)	41.23 (35.52–46.95)	< 0.001

*OP*, osteoporosis, *BMI* body mass index, *SMI* skeletal muscle index

The area under the curve (AUC) predicted by the SMI model for osteoporosis in Institution 1 patients was 0.572 (*p* = 0.003 95% CI 0.526–0.619), the AUC predicted by the SMI model for osteoporosis in Institution 2 patients was 0.644 (*p* = 0.038 95% CI 0.569–0.718), the AUC predicted by the SMI model for osteoporosis in Institution 3 patients was 0.589 (*p* = 0.032 95% CI 0.509–0.668) and the AUC predicted by the SMI model for osteoporosis in Institution 4 patients was 0.689 (*p* < 0.001 95% CI 0.589–0.789). Supplementary Fig. S1 showed the ROC results of Institution 1, Institution 2, Institution 3, and Institution 4 SMI in predicting osteoporosis, respectively.

### Performance of the CNN model

**Fig. 3:**
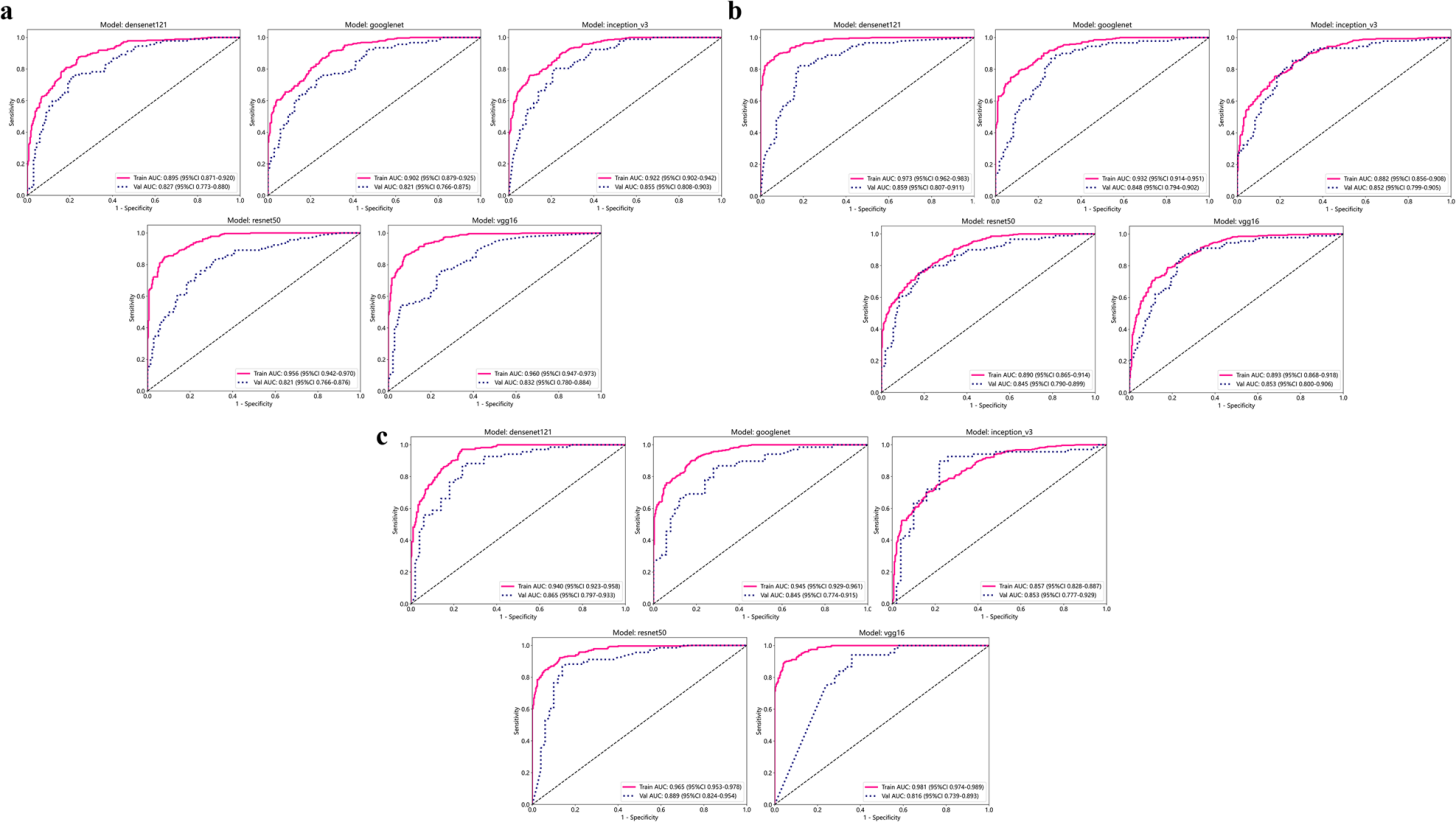
Receiver operating characteristic curves of the CNN model for (**a**) external test set 1, (**b**) external test set 2, and (**c**) external test set 3. CNN, convolutional neural network

In this study, five CNN models were constructed, namely Densent121, Inception_v3, Googlenet, Resnet50, and VGG16. In the training set based on the Institution1 data set, the five CNN models achieved good performance in predicting osteoporosis patients in different external test sets. The Densenet121 model achieved AUC scores of 0.827, 0.859, and 0.865 on external test sets 1, 2, and 3, respectively. The Inception_v3 model showed AUC scores of 0.855, 0.852, and 0.853 on the same sets. The Googlenet model’s AUC scores were 0.821, 0.848, and 0.845, while the Resnet50 model’s scores were 0.821, 0.845, and 0.889. Lastly, the Vgg16 model had AUC scores of 0.832, 0.853, and 0.816 on external test sets 1, 2, and 3, respectively (details are shown in Table 2 and Fig. 3). Of all the CNN models, Densenet121 has the highest average F1 score of 0.865 across the three external test sets. Therefore, the Densenet121 model has been selected for the subsequent study.

**Table 2:** Performance comparison of different CNN models in three external test sets

		AUC (95% CI)	Accuracy	Sensitivity	Specificity	F1-score
**External test set 1**						
Densenet121	Train	0.895 (0.871–0.920)	0.814	0.873	0.758	0.821
	Test	0.827 (0.773–0.880)	0.768	0.750	0.779	0.723
Inception_v3	Train	0.922 (0.903–0.942)	0.831	0.757	0.902	0.814
	Test	0.855 (0.808–0.903)	0.789	0.793	0.787	0.753
Googlenet	Train	0.902 (0.879–0.925)	0.804	0.908	0.704	0.819
	Test	0.821 (0.879–0.925)	0.754	0.728	0.772	0.705
Resnet50	Train	0.956 (0.942–0.970)	0.883	0.845	0.919	0.876
	Test	0.821 (0.766–0.876)	0.741	0.815	0.691	0.718
Vgg16	Train	0.960 (0.947–0.974)	0.888	0.859	0.916	0.882
	Test	0.832 (0.780–0.884)	0.750	0.750	0.750	0.708
**External test set 2**						
Densenet121	Train	0.973 (0.963–0.983)	0.905	0.894	0.916	0.902
	Test	0.859 (0.808–0.912)	0.818	0.811	0.824	0.802
Inception_v3	Train	0.882 (0.856–0.908)	0.790	0.754	0.825	0.778
	Test	0.852 (0.799–0.905)	0.788	0.844	0.741	0.784
Googlenet	Train	0.932 (0.914–0.951)	0.840	0.789	0.889	0.828
	Test	0.848 (0.794–0.902)	0.793	0.878	0.722	0.794
Resnet50	Train	0.890 (0.865–0.914)	0.790	0.750	0.828	0.777
	Test	0.845 (0.790–0.900)	0.788	0.767	0.806	0.767
Vgg16	Train	0.893 (0.868–0.918)	0.802	0.782	0.822	0.794
	Test	0.853 (0.800–0.907)	0.798	0.856	0.750	0.794
**External test set 3**						
Densenet121	Train	0.940 (0.923–0.958)	0.862	0.968	0.761	0.873
	Test	0.865 (0.797–0.933)	0.814	0.853	0.760	0.841
Inception_v3	Train	0.857 (0.828-0.887)	0.769	0.697	0.838	0.747
	Test	0.853 (0.777-0.929)	0.839	0.882	0.780	0.863
Googlenet	Train	0.945 (0.929–0.961)	0.859	0.898	0.822	0.861
	Test	0.845 (0.774–0.915)	0.788	0.838	0.720	0.820
Resnet50	Train	0.965 (0.953–0.978)	0.895	0.919	0.872	0.895
	Test	0.889 (0.824–0.955)	0.856	0.853	0.860	0.872
Vgg16	Train	0.981 (0.974–0.989)	0.924	0.894	0.953	0.920
	Test	0.816 (0.739–0.893)	0.805	0.926	0.640	0.846

*AUC* area under the curve

**Fig. 4:**
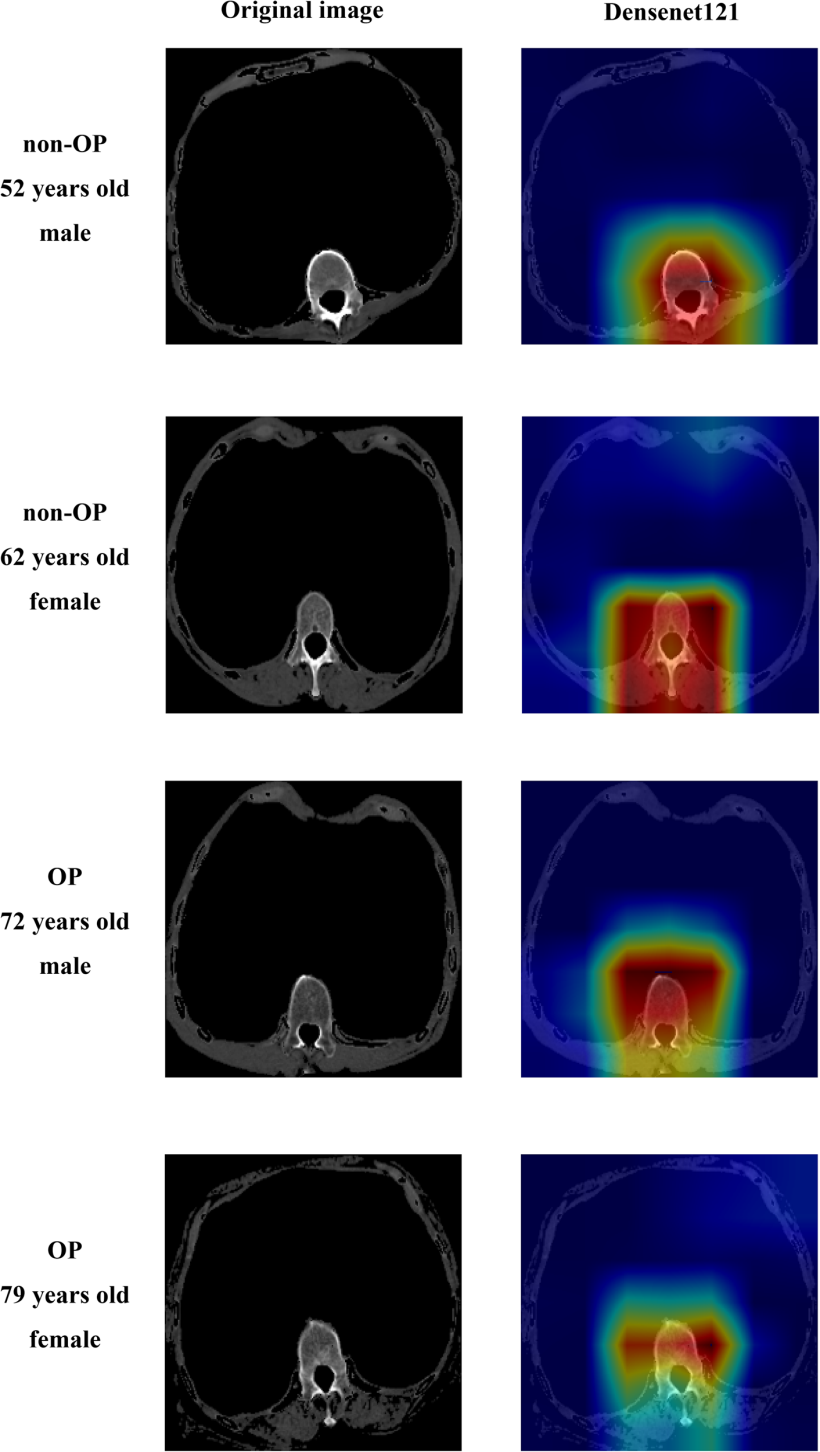
The focus areas of the Densenet121 model in the chest CT images. CT, computed tomography; OP, osteoporosis

Regarding model interpretability in male/female patients with and without osteoporosis, Densenet161’s areas of concern included the vertebral body and muscles (Fig. 4).

### Comparison of performance between CNN and SMI models

In order to further prove whether there are differences in the performance of the SMI model and the CNN model in predicting osteoporosis, this study conducted the Delong test on the AUC of the Densenet121 model and the SMI model. In both the training and external test sets, the Densenet121 model predicted that the AUC of osteoporosis was greater than that of the SMI model, and there were significant differences in all of them (Details are shown in Table 3).

**Table 3:** Predictive performance of various approaches in the osteoporosis training and external test sets

	AUC (95% CI)	Sensitivity	Specificity	*p*-value^a^
**Training set**				
SMI model	0.572 (0.526–0.619)	0.458	0.715	< 0.001
Densenet121	0.895 (0.871–0.920)	0.873	0.758	
**External test set 1**				
SMI model	0.644 (0.569–0.718)	0.859	0.402	< 0.001
Densenet121	0.827 (0.773–0.880)	0.750	0.779	
**External test set 2**				
SMI model	0.589 (0.509–0.668)	0.463	0.744	< 0.001
Densenet121	0.859 (0.808–0.912)	0.811	0.824	
**External test set 3**				
SMI model	0.689 (0.589–0.789)	0.460	0.926	0.004
Densenet121	0.865 (0.797–0.933)	0.853	0.760	

*AUC* area under curve, *SMI* skeletal muscle index

^a^ Comparison between Densenet121 and SMI model using the DeLong test

## Discussion

These five CNN models better predict osteoporosis, and their AUC in the external test set is more than 0.8. Of all the CNN models, Densenet121 has the highest average F1 score of 0.865 across the three external test sets. The AUC of the Densenet121 model in external test set 1, external test set 2, and external test set 3 were 0.827, 0.859, and 0.865, respectively. In addition, the Densenet121 model was significantly better than the SMI model in predicting osteoporosis patients. Therefore, our team chose the Densenet121 model for the follow-up study.

Densenet121 belongs to the DenseNet family and is an architecture of CNNs in deep learning, often used for image classification tasks. Densenet121 can effectively identify chest films with abnormal diseases, such as emphysema [17]. In addition, the Densenet121 model can identify cataract patients in time through images of the fundus [18]. Similarly, based on CT images of vertebral bodies, the Densenet121 model can better identify osteoporosis patients [19].

Many previous studies have shown that skeletal muscle and bone tissue are closely related. There is bidirectional regulation between muscle and bone, which includes mechanical interactions and endocrine interlinkages, all of which play a role in bone and muscle homeostasis [20]. In addition, sarcopenia and osteoporosis are also degenerative diseases of old age, and the elderly often suffer from two chronic diseases: sarcopenia and osteoporosis [21]. Kajiki Y et al [22] found that SMI can predict osteoporosis and fracture risk to some extent.

However, these studies have limitations, such as small sample size and moderate diagnostic performance. Therefore, this study constructed a Densenet121 model to predict osteoporosis based on the skeletal muscle and vertebral body images of unenhanced chest CT from four institutions and found that the prediction performance of the Densenet121 model was significantly higher than that of the SMI model.

Grad-CAM is a visualization technique used to understand which regions of an image are most important for a model’s predictions [15]. It’s a way to make the model’s decision-making process more transparent, which is particularly useful in deep learning models where the inner workings can often be complex and opaque. Grad-CAM helps to demystify the “black box” nature of deep learning models by showing which parts of the input image the model is focusing on to make its predictions. In this study, the Grad-CAM algorithm was used to explore the focus area of the Densent121 model in the process of patient classification. As our team suspected, the Densenet121 model screens osteoporosis patients by focusing on bone and skeletal muscle tissue in chest CT rather than bone or skeletal muscle tissue alone.

With the popularity of CT equipment, more and more patients will have unenhanced chest CT examinations. Clinical guidelines state that annual chest CT screening for lung cancer is recommended for people over 50 years of age [23]. In order to better assess the risk of surgery in middle-aged and elderly patients, clinicians tend to use chest CT to assess the cardiorespiratory function of these patients [24, 25]. Therefore, this study made secondary use of these CT images to build a CNN model to screen out patients with osteoporosis in advance, and timely anti-osteoporosis intervention was conducted for these patients to prevent the occurrence of osteoporotic fractures.

Opportunistic screening for osteoporosis during routine medical examinations or while investigating other conditions can substantially reduce the cost and complexity of screening programs. By leveraging unenhanced chest CT scans, which are increasingly common for various indications, our study capitalized on this approach to screen for osteoporosis. This method not only improves the efficiency of healthcare resources but also has the potential to enhance the quality of life for patients by enabling earlier detection and intervention. The use of CNN models like Densenet121 allows for a more accurate and timely diagnosis, which is crucial for preventing osteoporotic fractures and associated complications. The opportunistic use of AI in screening also opens avenues for broader research and clinical applications. It encourages the development of more inclusive screening strategies that can be integrated into existing healthcare practices without imposing additional burdens on patients or healthcare systems.

However, there are some limitations to this study. First, this study is a retrospective study, which may lead to selection bias. Future prospective cohort studies are needed to validate the model of this study further. Secondly, this study’s CNN model applies to men over 50 and postmenopausal women and does not have universal applicability across all age groups. Third, this study model only screened patients with osteoporosis and did not screen patients with bone loss.

## Conclusion

The CNN model based on unenhanced chest CT vertebral and skeletal muscle images can opportunistically screen patients with osteoporosis. Clinicians can use the CNN model to intervene in patients with osteoporosis and promptly avoid fragility fractures.

## Supplementary information

ELECTRONIC SUPPLEMENTARY MATERIAL

## Data Availability

The corresponding author can provide all the data sets used in this work upon reasonable request.

## Abbreviations

AUC: Area under curve
BMD: Bone mineral density
BMI: Body mass index
CNN: Convolutional neural network
CT: Computed tomography
DXA: Dual-energy X-ray absorptiometry
Grad-CAM: Gradient-weighted class activation mapping
OP: Osteoporosis
ROC: Receiver operating characteristic
ROI: Region of interesting
SMI: Skeletal muscle index
