# Supplementary material for: Application of deep learning model based on unenhanced chest CT for opportunistic screening of osteoporosis: a multicenter retrospective cohort study
Source: Insights Imaging. 2025 Jan 10;16:10. doi: 10.1186/s13244-024-01817-2 (PMC11723875; doi:10.1186/s13244-024-01817-2)
Supplement: Supplementary file 1 — ELECTRONIC SUPPLEMENTARY MATERIAL [file 13244_2024_1817_MOESM1_ESM.pdf]

Application of deep learning model based on unenhanced chest CT for opportunistic screening of osteoporosis: A multicenter retrospective cohort study

ELECTRONIC SUPPLEMENTARY MATERIAL

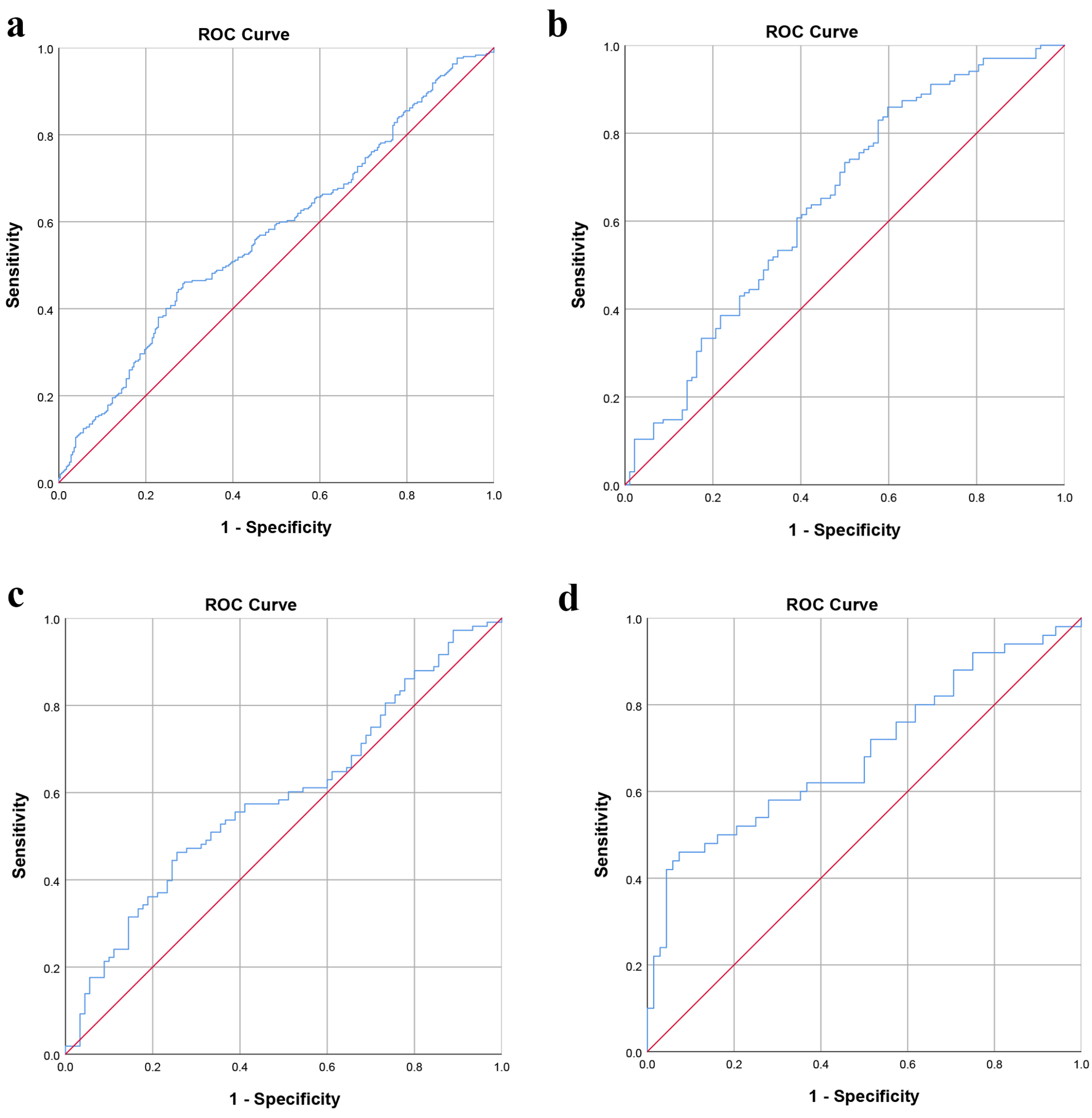

Supplementary Fig. S1, Receiver operating characteristic curves of SMI model for a) Institution 1, b) Institution 2, Institution 3 and Institution 4.

Abbreviations SMI, skeletal muscle index
